# Factors associated with phylogenetic clustering of hepatitis C among people who inject drugs in Baltimore

**DOI:** 10.1186/s12879-020-05546-x

**Published:** 2020-11-10

**Authors:** Oluwaseun Falade-Nwulia, Jada Hackman, Shruti H. Mehta, Sean D. McCormick, Gregory D. Kirk, Mark Sulkowski, David Thomas, Carl Latkin, Oliver Laeyendecker, Stuart C. Ray

**Affiliations:** 1 Johns Hopkins University School of Medicine, Baltimore, MD 21205 USA; 2 Johns Hopkins University Bloomberg School of Public Health, Baltimore, MD 21205 USA

**Keywords:** Hepatitis C, Molecular epidemiology, People who inject drugs, HIV, Women

## Abstract

**Background:**

The availability of effective, oral direct acting antivirals (DAAs) for hepatitis C virus (HCV) treatment has put elimination of HCV as a public health challenge within reach. However, little is known about the characteristics of transmission networks of people who inject drugs (PWID).

**Methods:**

Sequencing of a segment of the HCV genome was performed on samples collected from a community-based cohort of PWID between August 2005 and December 2016. Phylogenetic trees were inferred, and clusters were identified (70% bootstrap threshold; 0.04 maximum genetic distance threshold). We describe sex, race, age difference, and HIV infection status of potential transmission partners. Logistic regression was used to assess factors associated with being in an HCV cluster.

**Results:**

Of 508 HCV genotype 1 viremic PWID, 8% (*n* = 41) were grouped into 20 clusters, consisting of 19 pairs and 1 triad. In adjusted analyses, female sex (odds ratio [OR] 2.3 [95% confidence interval (CI) 1.2–4.5]) and HIV infection (OR 5.7 [CI 2.7–11.9]) remained independently associated with being in an HCV infection cluster.

**Conclusions:**

Molecular epidemiological analysis reveals that, in this cohort of PWID in Baltimore, HIV infection and female sex were associated with HCV clustering. Combination HCV prevention interventions targeting HIV infected PWID and addressing HCV infection prevention needs of women have potential to advance HCV elimination efforts.

**Supplementary Information:**

The online version contains supplementary material available at 10.1186/s12879-020-05546-x.

## Introduction

Hepatitis C virus (HCV) infection is a major public health challenge that disproportionately affects people who inject drugs (PWID) with a prevalence of 50–80% [1]. Shared use of drug preparation equipment (including cotton, cookers, and water) is the major route of HCV transmission among PWID [2]. This results in persistently high HCV transmission rates among PWID, with annual incidence rates ranging from 3 to 66 infections/100 person-years [3–6]. Recent increases in injection opioid use have further escalated HCV incidence and contributed to increased mortality due to HCV-related conditions [7, 8].

The availability of effective, oral direct acting antivirals (DAAs) for HCV treatment has fueled optimism for HCV elimination, including a call by the World Health Organization for the elimination of HCV as a public health challenge by 2030 [9, 10]. Identifying characteristics of individuals in HCV transmission networks would provide critical information for the development and implementation of targeted HCV treatment interventions.

Phylogenetic analyses have been used to document transmission of both HIV and HCV infections between partners and within social networks [11–14]. Recent phylogenetic analyses suggested dense networks of HIV transmission in a rural community in Indiana, United States linked to injection use of oxymorphone [12]. This analysis also demonstrated that HIV spreads rapidly through existing HCV transmission networks [15]; highlighting the importance of understanding HCV transmission networks for prevention of HCV and HIV transmission among PWID. Less is known about HCV transmission networks in urban areas of the United States.

In this study, we sought to examine phylogenetic clustering, cluster composition and factors associated with clustering among participants enrolled in the AIDS Linked to the Intravenous Experience (ALIVE) cohort, a longstanding prospective cohort of current and former PWID followed in Baltimore, Maryland, United States.

## Methods

### Study population and design

Data and specimens for this study were from participants enrolled in the ALIVE study, a prospective community-recruited cohort of former and current PWID residing in Baltimore, Maryland [16]. The cohort was initially recruited in 1988 and then replenished with additional recruitment during 1994–1995, 1998, and 2000, 2005–2008, and 2015–2016. Participants are assessed at study entry and at subsequent semi-annual visits with collection of sociodemographic, behavioral, and clinical information; performance of clinical examinations; and biological specimen collection. All participants in ALIVE have received at least one HCV antibody test at entry into the study. Periodically, HCV antibody positive participants have an HCV RNA assessment. In 2016, we conducted HCV RNA testing on all participants currently in follow-up; for those who were no longer in follow-up, we tested HCV RNA from their most recent visit after 2005. This analysis was restricted to participants who had a hepatitis C viremic specimen available for HCV RNA testing and subsequent sequencing.

### HCV RNA testing and sequencing

The most recent study sample among HCV antibody positive ALIVE participants in follow up between August 2005 and December 2016 was quantified for HCV RNA using a quantitative reverse transcription polymerase chain reaction (RT-PCR) assay (TaqMan HCV analyte-specific reagent; Roche Molecular Diagnostics, Indianapolis, IN) with the DNA amplification products monitored on a COBAS TaqMan Analyzer (Roche Molecular Diagnostics, Indianapolis, IN). This assay has a lower limit of detection of 50 IU/mL. Sequencing was attempted on all samples with detectable HCV RNA. HCV sequences spanning the majority of the Core-E1 region were amplified from viremic specimens following total RNA extraction using a QIAamp MinElute Virus Spin column (Qiagen, Valencia, CA) according to the manufacturer’s instructions. Direct sequencing of RT-PCR products from the Core-E1 region was performed as previously described [17]. Samples not successfully amplified initially were retested.

### Phylogenetic analyses

Reference sequences were retrieved from GenBank through a BLAST search for HCV sequences similar to study sequences in order to support rigorous identification of “local clusters”. To further assess the performance of our clustering method, longitudinal samples were retrieved from PubMed using a cohort of 10 women infected with HCV genotype 1b through treatment with contaminated anti-D immune globulin (Irish anti-D cohort) [18]. These sequences (supplementary data), in addition to an HCV genotype 1c sequence (included for outgroup-rooting), were aligned and analyzed with RAxML version 8.2 [19] using the GTRGAMMA model with 20 independent reconstructions and rapid maximum likelihood bootstrap with 1000 iterations. Trees were rendered using Dendroscope version 3.6.3 [20]. Phylogenetic trees were inferred for HCV subtype 1a and 1b separately. Nucleotide substitution model selection was based on the corrected Akaike information criterion scores of various models [21].

The final fragment analyzed was 342 base pair long following the removal of positions containing gaps. Clusters were identified using ClusterPicker software [22]. Sensitivity analyses were performed by varying the genetic distance threshold between 0.025–0.065 and removing bootstrap support at 0.04 genetic distance threshold to determine the effect on identification of factors associated with clustering (Supplementary Table 1).

### Study outcome

The primary outcome was phylogenetic clustering of HCV infections, defined as ≥2 participants with HCV genome sequences satisfying a 70% bootstrap and 0.04 maximum genetic distance threshold requirement for sequence similarity similar to previous analyses of phylogenetic clustering conducted in this cohort [23] . Those meeting these criteria were considered potential transmission partners.

### Statistics

Descriptive statistics were used to characterize the study population with respect to demographics and risk behaviors. Proportions were compared using chi-squared tests. We analyzed cluster composition to understand the differences in sex, race, age, and HIV infection status of potential transmission partners. We calculated the percentage of PWID within clusters who were of the same or different sex, race or HIV infection status, and for age, transmission partners with a less than or 10 year or greater difference in age. Univariable and multivariable logistic regression analyses were used to determine odds ratios for factors associated with being in an HCV infection cluster. Factors were considered for inclusion in multivariable analysis if they demonstrated an association with the outcome at the level of *p* < 0.1 in univariable analysis. Analyses were performed using Stata version 13 (Stata Corp, College Station, Texas).

The study was conducted in accordance with provisions of the Declaration of Helsinki. It was approved by the Johns Hopkins Bloomberg School of Public Health Institutional Review Board. All participants provided informed consent prior to inclusion in this study.

## Results

### Study population characteristics

A total of 2312 PWID were enrolled in the ALIVE cohort between 2005 and 2016 of which 1873 (81%) were HCV antibody positive. Of 1795 PWID tested for HCV RNA, 1311 (73%) had a detectable HCV RNA sample of which, 600 (48%) had samples sequenced and were included in this study. The Core-E1 region was amplifiable in 100%, with good quality sequence obtainable in 566 participants (88%). Of these, 510 (90%) were HCV genotype 1 sequences. An additional 2 sequences did not have associated participant demographic data and were excluded from analyses of factors associated with clustering.

Among the 508 participants included in the analysis, the median age was 54 years (Interquartile range [IQR] 47–59), majority were male (68% [345/508]), black (87% [441/508]) and infected with HCV genotype 1a infection (83% [421/508]) (Table 1). The mean duration of injection drug use was 13 years (IQR 2–24). Approximately a third (33% [167/508]) of participants were HIV infected at enrollment into ALIVE and an additional 29 participants (6%) seroconverted to HIV positive during follow-up, leading to an overall HIV coinfection prevalence of 39% at most recent testing. Among HIV infected participants, 57% (111/196) had an HIV RNA < 400 copies/ml, 26% (51/196) had an HIV RNA ≥ 400 copies/mL, and 17% (34/196) had missing HIV RNA data at the time of sample collection for HCV sequencing.

**Table 1:** Characteristics of participants with HCV Core-E1 sequences in the ALIVE cohort

Characteristic	Total (%) (***n*** = 508)
Age, years (median [IQR])	53.9 (46.8–59.3)
Black race	441 (86.8)
Male sex	345 (67.9)
Female sex	163 (32.1)
High school education or higher	202 (39.8)
Homelessness^a^	98 (19.3)
Incarcerated ≥1 week^a^	51 (10.1)
HCV Genotype	
1a	421 (82.9)
1b	87 (17.1)
HIV positive at baseline	167 (32.9)
HIV seroconverted (baseline to most recent visit)	29 (5.7)
HIV positive at most recent visit	196 (38.6)
Most recent HIV RNA among HIV infected^b^	
HIV RNA < 400 copies/ml	111 (56.6)
HIV RNA ≥ 400 copies/ml	51 (26.0)
Prescribed methadone or buprenorphine	176 (34.7)
Injection drug use behaviors	
Years injecting (mean [SD])	12.68 (10.8)
Ever shared a syringe at baseline	451 (89.1)
Injected drugs^a^	204 (40.2)
Shared syringe^a^	85 (16.7)
Used crack^a^	157 (31.0)
Injected or snorted cocaine^a^	112 (22.1)
Injected heroin^a^	146 (28.7)
Snorted heroin^a^	85 (16.7)
Injected speedball^a^	122 (24.0)
Visited SSP in the last month	61 (12.1)

^a^Within 6 months of visit

^b^HIV RNA unavailable in 34 HIV infected patients

*HCV* hepatitis c virus, *SSP* syringe service program, *HIV* human immunodeficiency virus, *RNA* ribonucleic acid, *IQR* interquartile range, *SD* standard deviation

At the time of sample collection, 40% (204/508) of participants reported injection drug use in the preceding 6 months, with almost half (85/204) of these reporting syringe sharing. In this group, the most frequently injected drug was heroin and only a minority (61/508) reported visiting a syringe service program in the preceding month.

### Phylogenetic cluster composition

**Fig. 1:**
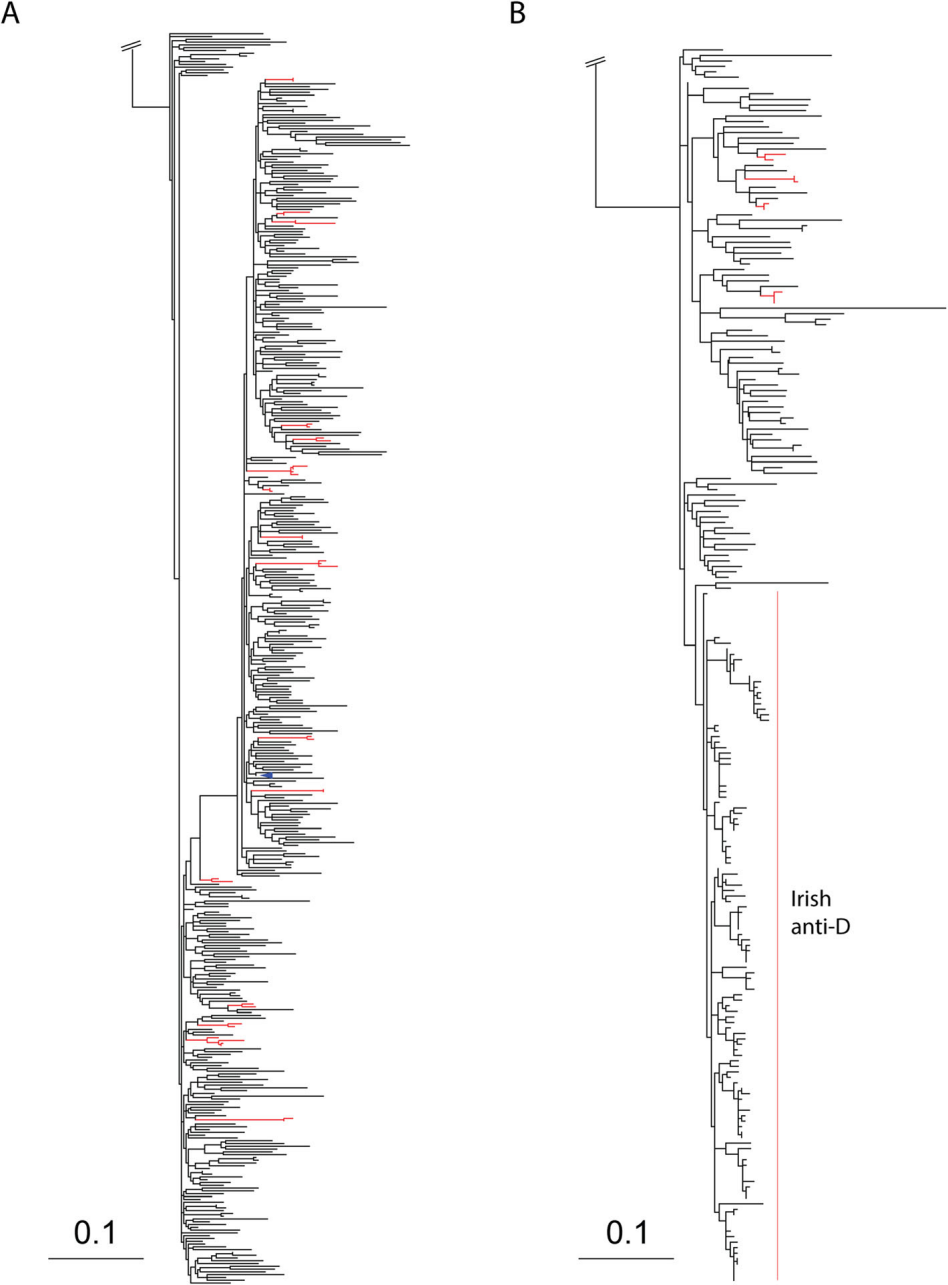
Phylogenetic trees of HCV (**a**) genotype 1a and (**b**) genotype 1b in the ALIVE Cohort, 2005–2016, Baltimore, Maryland, USA. The maximum likelihood trees were inferred using RAxML version 8.2 and participants in clusters (red) differentiated from non-clustered participants (black) using ClusterPicker with a bootstrap threshold of 70% and a genetic distance of 0.04

**Fig. 2:**
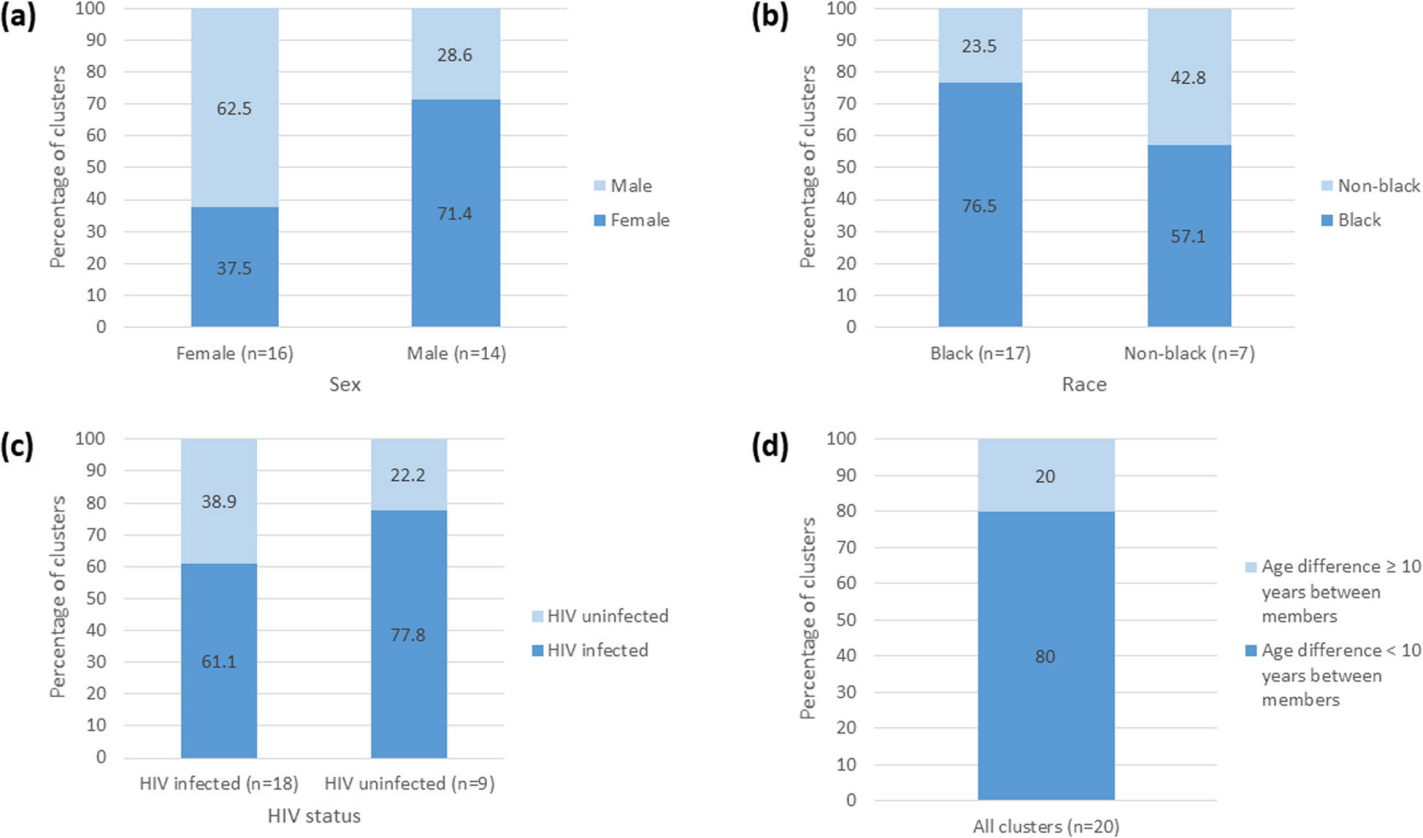
(**a**-**c**) Proportion of clusters by (**a**) sex, **b** (race) and (**c**) HIV status among clusters with at least one member fulfilling each characteristic. **d** Proportion of clusters with and without an age difference > 10 years between members

Phylogenetic trees of the core to E1 region of HCV are shown for genotypes 1a (*n* = 421) and 1b (*n* = 87) in Fig. 1a and b, respectively. Among the 508 participants included in this study, 41 participants were grouped either in a pair (*n* = 38) or triad (n = 3). The majority of clusters were among samples collected in 2016 with a median of 298 days between when samples in the same clusters were collected. We observed disassortativity by sex with clusters including at least 1 female participant having a male potential transmission partner in the majority of cases (10 of 16 clusters (62.5%) and a female potential transmission partner in the minority of cases (6 of 16 clusters (37.5%) (Fig. 2a). Among black and non-black participants, most participants had potential transmission partners that were black (13 of 17 clusters (76.5%) in clusters with at least 1 black participant and 4 of 7 clusters (57.1%) with at least 1 non-black participant) (Fig. 2b). In clusters with at least 1 HIV infected participant, the majority of potential transmission partners were HIV infected (11 of 18 clusters (61.1%). Similarly, in clusters with at least 1 HIV uninfected participant the majority of potential transmission partners were HIV infected (7 of 9 clusters (77.8%) (Fig. 2c). We observed assortativity by age with less than a 10 year age difference between majority of participants in the same cluster (Fig. 2d).

There were 14 clusters among 73 longitudinal samples from 10 individuals in the Irish anti-D cohort, included in the genotype 1b phylogenetic tree. Six of these clusters were composed of longitudinal samples from 6 individuals (1 individual per cluster) and 8 clusters composed of longitudinal samples from 4 individuals (2 clusters per individual).

### Univariable analysis of factors associated with phylogenetic clustering

In unadjusted logistic regression analyses, membership in a cluster was significantly associated with younger age (per 10 year age decrease) (Odds ratio [OR] 1.59 [95% Confidence interval (CI) 1.15–2.20]), female sex (OR 2.98 [CI 1.56–5.70]), HIV infection (OR 4.95 [CI 2.42–10.12]), and non-black race (OR 2.69 [CI 1.28–5.67]) (Table 2).

**Table 2:** Logistic regression analysis of factors associated with being in a phylogenetic cluster for participants with Genotype 1a or 1b in the ALIVE cohort

Characteristic	Total (%) (***n*** = 508)	Not in Cluster (%) (***n*** = 467)	In Cluster (%) (***n*** = 41)	Univariate	***P***-value	Multivariate	***P***-value
Age (per 10 year decrease)	–	–	–	1.59 (1.15, 2.20)	**< 0.01**	1.57 (0.98, 2.53)	0.06
Female Sex	163 (32.1)	140 (30.0)	23 (56.1)	2.98 (1.56, 5.70)	**< 0.01**	2.32 (1.19, 4.55)	**0.01**
Race							
Black	441 (86.8)	411 (88.0)	30 (73.1)	Ref.	–	Ref.	–
Non-black	67 (13.2)	56 (12.0)	11 (26.8)	2.69 (1.28, 5.67)	**< 0.01**	2.42 (0.95, 6.21)	0.07
HCV Genotype							
1a	421 (82.9)	388 (83.1)	33 (80.5)	Ref.	–	–	–
1b	87 (17.1)	76 (16.9)	8 (19.5)	1.70 (0.82, 3.51)	0.15	–	–
HIV positive	196 (38.6)	166 (35.5)	30 (73.2)	4.95 (2.42, 10.12)	**< 0.01**	5.67 (2.70, 11.92)	**< 0.01**
High school education or higher	202 (39.8)	184 (39.5)	18 (43.9)	1.20 (0.63, 2.28)	0.58	–	–
Homelessness^a^	98 (19.3)	91 (19.5)	7 (17.1)	0.85 (0.37, 1.98)	0.71	–	–
Shared a syringe^a^	85 (16.7)	(79 16.9)	6 (14.6)	0.84 (0.34, 2.07)	0.71	–	–
Visit SSP in last month	61 (12.1)	57 (12.5)	3 (7.3)	0.55 (0.17, 1.85)	0.34	–	–
Injection drug use behaviors^a^							
Any injection drug use	204 (40.2)	189 (40.6)	15 (36.6)	0.85 (0.44, 1.64)	0.62	–	–
Use crack	157 (31.0)	146 (31.3)	11 (26.8)	0.80 (0.39, 1.65)	0.55	–	–
Inject cocaine	112 (22.0)	103 (22.1)	9 (22.0)	1.03 (0.46, 2.32)	0.94	–	–
Inject heroin alone	146 (28.7)	137 (29.3)	9 (22.0)	0.68 (0.31, 1.46)	0.32	–	–
Inject speedball	122 (24.0)	114 (24.4)	8 (19.5)	0.75 (0.34, 1.67)	0.48	–	–
Year sample collected							
2005–2009	99 (19.5)	93 (19.9)	6 (14.6)	Ref.	–	Ref.	–
2010–2014	48 (9.5)	42 (9.0)	6 (14.6)	2.21 (0.67, 7.30)	0.19	1.07 (0.31, 3.76)	0.91
2016	361 (71.1)	332 (71.1)	29 (70.7)	1.35 (0.53, 3.36)	0.51	2.48 (0.88, 6.99)	0.09

^a^Within 6 months of visit

*HCV* hepatitis c virus, *HIV* human immunodeficiency virus, *SSP* syringe service program

### Multivariable analyses of factors associated with phylogenetic clustering

In multivariable analyses that adjusted for year of sample collection, female sex (OR 2.32 [CI 1.19–4.55]), and HIV infection (OR 5.67 [CI 2.70–11.92] remained independently associated with being in a cluster of related HCV infections (Table 2).

## Discussion

This study characterizes phylogenetic clustering of HCV in a cohort of urban PWID recruited in Baltimore, Maryland, United States. Among these PWID with samples collected over a period spanning 2005 to 2016, we found evidence of clustering suggestive of potential transmission events in only 8% (*n* = 41) of participants. Clustering was associated with female sex, and HIV co-infection. We found evidence of disassortative mixing by sex. We also found evidence of racial assortative mixing among participants of black race and disassortative mixing by HIV status among HIV uninfected participants. These findings have important implications for targeting of public health interventions aimed at preventing the spread of both HCV and HIV infections.

HIV infection was independently associated with being in a cluster in this study. This association of HIV infection with HCV clustering has previously been described [24, 25] and suggests that individuals with HIV/HCV co-infection represent a group at greater risk of behaviors leading to onward HCV transmission, for which targeted interventions, including HCV treatment for prevention of transmission, could provide disproportionate benefit [24–27]. Of note, in this group of HIV infected PWID, a significant proportion had detectable HIV viremia and only a minority reported accessing syringe service programs. Although HCV is approximately 10 times more transmissible than HIV [28], continued high-risk injection practices could also result in HIV transmission, as noted in the recent HIV transmission outbreak in Indiana [15]. This is especially concerning given the high proportion of HIV uninfected individuals who had potential HCV transmission partners who were HIV infected. Our findings reinforce the need for interventions to increase uptake of harm reduction interventions, including syringe service programs, medication assisted treatment, frequent testing for both HIV and HCV, and immediate treatment to prevent and break transmission cycles for both HIV and HCV among PWID [29–32].

We also found female sex to be associated with HCV clustering. Increased HCV clustering in females may be linked to differences in the way women experience substance use compared to men. Women are more likely to have a sexual partner facilitate their initiation of injection drug use by guiding or administering their first injection [33–36]. Further, individuals who are injected by someone else at initiation are more likely to report receptive syringe and other equipment sharing at initiation and within the past 6 months [37, 38]. Sexual relationships have also been associated with increased sharing of drug use paraphernalia [39]. Male sexual partners may particularly influence injection practices of women [33, 34, 40]. It is thus not surprising that a higher risk of incident HCV infection has been documented in women compared to men in the setting of heterosexual relationships in another study [41]. While our analysis cannot confirm direct transmission, existing sexual relationships, or information on behaviors at injection initiation, most women who were part of a cluster in our analysis clustered with men, which is supportive of our hypothesis of transmission of HCV through injection drug use with individuals that may also be sexual partners. Interventions taking into account the experiences of women who use drugs are needed to reduce rates of HCV infection and reinfection in female PWID, especially given recent increases in incident HCV infection among women [42].

Overall rates of HCV clustering found in our study were lower than those of 22–37% reported in other studies, including one from this cohort [23–25, 43–45]. This study differs from the previous study evaluating clustering of HCV in the ALIVE cohort in that samples were sequenced from earlier in the HCV epidemic (1988) at a time when majority of the participants were younger (median age < 35 years compared to the median age of 53 years of participants in this current study) suggestive of shorter duration of HCV infection. Lower rates of HCV infection clustering in our study is likely due to the long duration of HCV infection in most individuals sampled in this analysis. The hepatitis C virus uses an error prone polymerase to replicate to high viral loads resulting in the generation in each individual host infected with HCV, genetically distinct viral variants. This, in addition to the impact of host immune response on viral evolution within individuals, will make it challenging to uncover infections from a common source occurring many years earlier [46, 47]. Our sensitivity analyses revealed intra but not inter person clustering of HCV sequences over time among sequences from women in the anti-D cohort, who were known to have been infected from a common source exposure to HCV, suggesting that clustering as defined in this analysis represents more recent transmission events. Low rates of clustering found in our study may also reflect differences in recruitment across studies, sampling density, or methods used for phylogenetic and cluster analyses. In the ALIVE cohort, the study population is recruited in discrete waves with targeted efforts to recruit a geographically diverse cohort that is representative of the estimated 19,000 people who use drugs in Baltimore [48]. It is thus possible that a low sampling density of HCV infected PWID may have reduced the sensitivity of finding evidence of clustering [49]. Additionally, most analyses have used a longer fragment of the HCV genome; for example, the Core to E2 region (1142 nucleotides), which is much longer than the Core to E1 region (342 nucleotides) used in our analyses, thus the latter may have lower sensitivity of cluster detection. However, the sensitivity of sequence recovery of the Core E1 amplicon (over 90% of HCV RNA positive samples are successfully sequenced) over that of longer amplicons, may balance the short sequence length by generating less missing sequence data for participants and has identified clustering from samples collected closer to infection [23].

Our study is limited by being restricted to a phylogenetic analysis of a sample of PWID enrolled in the ALIVE cohort, which may not be representative of all PWID in Baltimore or in other urban areas of the United States. Additionally, these analyses cannot determine all linked transmissions, as there are likely individuals who were not participants in this study, but may have acquired HCV and then transmitted to others as components of transmission networks. We also do not have information on potential social relationships between individuals identified as being in a cluster. Our analyses do, however, identify factors associated with being in a cluster suggestive of linked transmission.

## Conclusion

In the era of availability of effective tools, such as oral DAA HCV therapies, syringe service programs, and medication-assisted treatment, with the potential to reduce HCV transmission, molecular epidemiology data such as these analyses, provide critical information to guide effective public health interventions to control HCV. Our study highlights the potential role of combination interventions targeting HIV infected PWID and addressing HCV infection prevention needs of women to reduce HCV infection transmission.

## Supplementary Information

**Additional file 1: Supplementary Table 1:** Logistic regression analysis of factors associated with being in a pair/cluster by increasing genetic distance threshold using ClusterPicker software. **Supplementary data:** Accession numbers of Irish anti-D cohort sequences included in analyses.

## Data Availability

The datasets used during the current study are available from the corresponding author on reasonable request.

## Abbreviations

DAAs: Direct acting antivirals
HCV: hepatitis c virus
PWID: people who inject drugs
ALIVE: AIDS Linked to the Intravenous Experience
RT-PCR: reverse transcription polymerase chain reaction
OR: Odds Ratio
CI: 95% Confidence Interval
