# Supplementary material for: Factors associated with phylogenetic clustering of hepatitis C among people who inject drugs in Baltimore
Source: BMC Infect Dis. 2020 Nov 10;20:815. doi: 10.1186/s12879-020-05546-x (PMC7652590; doi:10.1186/s12879-020-05546-x)
Supplement: Supplementary file 1 — Additional file 1: Supplementary Table 1: Logistic regression analysis of factors associated with being in a pair/cluster by increasing genetic distance threshold using ClusterPicker software. Supplementary data: Accession numbers of Irish anti-D cohort sequences included in analyses. [file 12879_2020_5546_MOESM1_ESM.docx]

**Factors associated with phylogenetic clustering of Hepatitis C among people who inject drugs in Baltimore**

**Supplementary Table and data**

| **Supplementary Table 1: Logistic regression analysis of factors associated with being in a pair/cluster by increasing genetic distance threshold using ClusterPicker software.** | | | | | | | | | | |
| --- | --- | --- | --- | --- | --- | --- | --- | --- | --- | --- |
| **Number of cluster** | **Genetic distance threshold** | | | | | | | | | |
|  | **0.025** | | **0.04** | | **0.04 no boot strap** | | **0.055** | | **0.065** | |
|  | **19 pairs** | | **19 pairs & 1 triad** | | **30 pairs, 3 triads, 4 tetrads** | | **19 pairs & 2 triads** | | **19 pairs & 3 triads** | |
| **Characteristic** | **Odds Ratio**  **(95% CI)** | **P-value** | **Odds Ratio**  **(95% CI)** | **P-value** | **Odds Ratio**  **(95% CI)** | **P-value** | **Odds Ratio**  **(95% CI)** | **P-value** | **Odds Ratio**  **(95% CI)** | **P-value** |
| Age (per 10 year decrease) | 1.58  (1.13, 2.21) | **0.008** | 1.59  (1.15, 2.20) | **0.005** | 1.37  (1.07, 1.76) | **0.010** | 1.54  (1.12, 2.11) | **0.008** | 1.54  (1.13, 2.09) | **0.006** |
| Female sex | 2.85  (1.46,5.57) | **0.002** | 2.98  (1.56, 5.70) | **0.001** | 1.52  (0.94, 2.46) | **0.088** | 2.81  (1.50, 5.24) | **0.001** | 2.93  (1.59, 5.38 | **0.001** |
| Race | | | | | | | | | | |
| Black | Ref. | - | Ref. | - | Ref. | - | Ref. | - | Ref. | - |
| Non-black | 2.59  (1.19, 5.61) | **0.016** | 2.69  (1.28, 5.67) | **0.009** | 1.69  (0.91, 3.13) | **0.095** | 2.43  (1.16, 5.08) | **0.018** | 2.53  (1.24, 5.17) | **0.011** |
| HIV positive | 5.03  (2.39, 10.62) | **0.000** | 4.95  (2.42, 10.12) | **0.000** | 1.71  (1.06, 2.72) | **0.026** | 4.88  (2.45, 9.73) | **0.000** | 4.31  (2.24, 8.28) | **0.000** |
| High school education or higher | 1.24  (0.64, 2.41) | 0.488 | 1.20  (0.63, 2.28) | 0.580 | 1.27  (0.79, 2.04) | 0.636 | 1.16  (0.622, 2.17) | 0.636 | 1.13  (0.62, 2.08) | 0.690 |
| Homelessness^A^ | 0.94  (0.40, 2.20) | 0.888 | 0.85  (0.37, 1.98) | 0.708 | 0.80  (0.43, 1.48) | 0.471 | 0.78  (0.33, 1.80) | 0.553 | 0.99  (0.46, 2.12) | 0.979 |
| Shared a syringe^A^ | 0.93  (0.38, 2.29) | 0.871 | 0.84  (0.34, 2.07) | 0.708 | 0.98  (0.52, 1.83) | 0.944 | 0.77  (0.31, 1.88) | 0.566 | 0.71  (0.29, 1.72) | 0.446 |
| Visited SSP^A^ | 0.61  (0.18, 2.03) | 0.417 | 0.55  (0.17, 1.85) | 0.338 | 0.84  (0.40, 1.78) | 0.649 | 0.51  (0.15, 1.70) | 0.273 | 0.66  (0.23, 1.90) | 0.436 |
| Injection drug behaviors^A^ | | | | | | | | | | |
| Current injection drug use | 0.97  (0.49, 1.90) | 0.921 | 0.85  (0.44, 1.64) | 0.619 | 0.88  (0.54, 1.42) | 0.594 | 0.75  (0.39, 1.44) | 0.386 | 0.83  (0.44, 1.54) | 0.551 |
| Used crack | 0.78  (0.37, 1.65) | 0.520 | 0.80  (0.39, 1.65) | 0.551 | 0.98  (0.59, 1.62) | 0.934 | 0.93  (0.47, 1.83) | 0.831 | 1.05  (0.55, 2.00) | 0.883 |
| Used cocaine | 1.11  (0.51, 2.41) | 0.800 | 0.99  (0.46, 2.15) | 0.988 | 1.19  (0.69, 2.06) | 0.517 | 0.90  (0.42, 1.94) | 0.790 | 1.09  (0.54, 2.22) | 0.814 |
| Injected heroin alone | 0.75  0.35, 1.64) | 0.475 | 0.68  (0.31, 1.46) | 0.319 | 0.97  (0.58, 1.63) | 0.910 | 0.61  (0.29, 1.31) | 0.208 | 0.74  (0.36, 1.49) | 0.398 |
| Injected speedball | 0.83  (0.37, 1.87) | 0.657 | 0.75  (0.34, 1.67) | 0.483 | 0.82  (0.47, 1.45) | 0.346 | 0.68  (0.31, 1.51) | 0.346 | 0.84  (0.41, 1.75) | 0.645 |
| ^A^Within 6 months of visit  SSP: syringe service program; HIV: human immunodeficiency virus | | | | | | | | | | |

**Supplementary data: Accession numbers of Irish anti-D cohort sequences included in analyses**

Accession numbers: JX649674-JX649683; JX649685-JX649687; JX649690; JX649691; JX649714; JX649719; JX649724; JX649727-JX649729; JX649731; JX649733; JX649736-JX649746; JX649749; JX649751-JX649755; JX649758; JX649759; JX649761-JX649765; JX649782-JX649783; JX649785; JX649787; JX649792; JX649794-JX649799; JX649818; JX649820; JX649822-JX649824; JX649828; JX649830; JX649832; JX649833; JX649835; JX649844-JX649847; JX649853) (18).
